# Identification of the aroma compounds in *Vitex doniana* sweet: free and bound odorants

**DOI:** 10.1186/s13065-017-0247-7

**Published:** 2017-02-23

**Authors:** Ola Lasekan

**Affiliations:** Department of Food Technology, University Putra Malaysia, 43400 Serdang, Malaysia

**Keywords:** *Vitex doniana* sweet, Free and bound volatile compounds, Odour activity values

## Abstract

**Background:**

Most often, the glycosidically-bound aroma compounds are released during industrial processing or pre-treatment of fruits. This usually introduces modification to the aroma notes of such fruits. Therefore, there is the need to understand the contribution of these bound aroma compounds to the overall aroma of a given fruit. In recent years research studies have reported on the free- and bound volatile compounds of several fruits. However, there is no report yet on *Vitex doniana* sweet.

**Results:**

Results of gas chromatography–mass spectrometry (GC–MS) and gas chromatography–olfactometry (GC–O) of free and glycosidically-bound aroma-active compounds from *Vitex doniana* sweet revealed a total of 35 compounds in the free fraction, and 28 compounds were in the bound fraction respectively. Whilst the major group of compounds in the free fraction were terpenes, alcohols, and esters, the bound fraction consisted of ketones, alcohols, terpenes and norisoprenoids.

**Conclusion:**

A comparative analysis of the aroma potencies of the free and bound volatile fractions revealed that; free fraction exhibited strong potency for the fruity and floral notes, and the bound fraction produced more of the flowery, caramel-like and cherry-like notes. In addition results of odour activity values showed that ethylbutanoate, β-damascenone, ethyl-2-methyl propionate, linalool, hexyl acetate and (*Z*)-rose oxide contributed highly to the sweet prune-like aroma of the fruit.

## Background

*Vitex doniana* sweet (Vds) is the edible fruit that belongs to the family *Lamiaceae*. There are about 250 species in this family [1]. *V. doniana* sweet is the most abundant and widespread of this genus in the Savannah regions. The fruit is commonly called ‘ucha koro’, ‘oori-nla’ and ‘mfudu’ or ‘mfulu’ in Swahili. *V. doniana* sweet is oblong, about 3 cm long. It is green when immature, and purplish-black on ripening with a starchy black pulp. Each fruit contains one hard conical seed which is about 1.5–2.0 cm long and 1–1.2 cm wide. The fruit which tastes like prunes is rich in nutrients including vitamins A (0.27 mg· 100^−1^g DB), B1 (18.33 mg· 100^−1^g DB), B2 (4.80 mg· 100^−1^g DB), B6 (20.45 mg· 100^−1^g DB) and C (35.58 mg· 100^−1^g DB) respectively [2]. The fruit which is consumed fresh can be made into jam and wine [3]. *V. doniana* sweet has a unique sweet prune-like aroma when ripened. Although, a number of sugars [4], amino acids and minerals [5] have been reported in Vds, however, there is no study yet on the components responsible for the unique sweet prune-like aroma of the Vds. Studies have shown that fruits’ aromatic components are either in the free form, or bound to sugar in the form of glycosides [6–8].

Most often, the glycosidically-bound aroma compounds are released during industrial processing or pre-treatment of fruits. This usually introduces modification to the aroma notes of such fruits [9]. Whilst several studies have reported on the free and glycosidically-bound volatiles in fruits such as strawberry [8], mango [10], raspberry [11], lychee [12], blackberry [6], acerola [7] and a host of other fruits, there has been no study on the volatile constituents of *Vitex doniana* sweet.

This study aimed at providing an insight into the free and glycosidically-bound aroma compounds of *Vitex doniana* sweet.

## Results and discussion

**Fig. 1:**
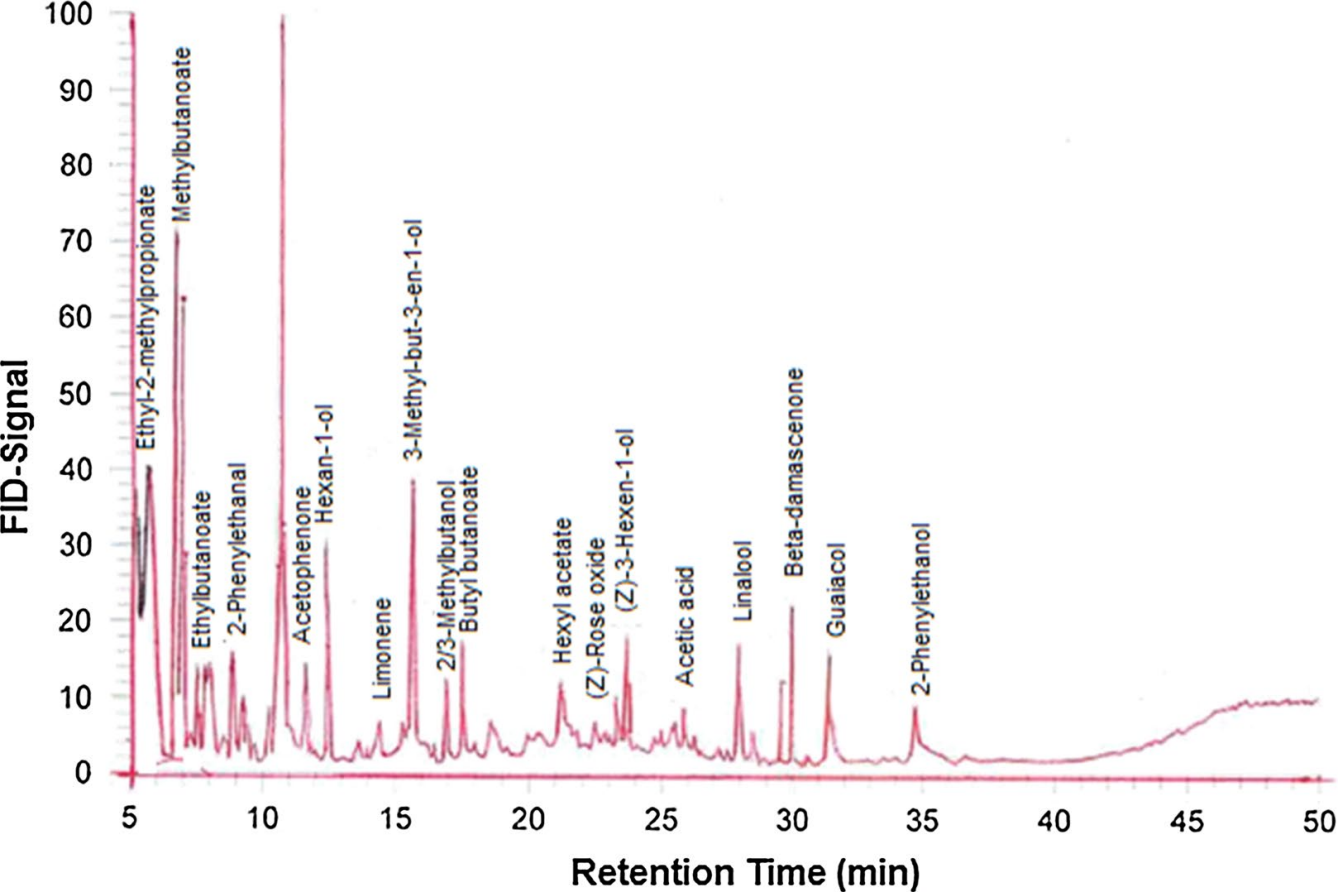
Characteristic gas chromatogram of solvent extracted sweet *Vitex doniana*

The volatile fractions of both free and glycosidically bound *V. doniana* sweet, separated on two columns (DB-FFAP and SE-54) of different polarity are shown in Table 1 and Fig. 1. A total of 35 compounds were identified in the free fraction while only 28 compounds were detected in the bound fraction. In general, the aroma compounds identified in both fractions were made up of alcohols (7), aldehydes (2), acids (2), esters (11), terpenes (9), ketones (3), norisoprenoids (7), and a phenol. The most important ones in terms of concentration and the numbers identified in the free fraction were the terpenes (43%), alcohols (29%), and esters (25%). On the other hand, in the bound fraction, the ketones, were the most abundant (29%) followed by the alcohols (26%), terpenes (20%) and the norisoprenoids (13%).

**Table 1:** The concentration of volatile compounds (free and bound) identified in *Vitex doniana* sweet (µg kg^−1^ of pulp)

Compounds^1^	LR1	LR2	Free	Bound
Alcohols				
3-Methyl-but-3-en-1-ol	1209	720	1046 ± 33.0^a^	570 ± 23.6^b^
2/3-Methyl-butanol	1213	738	153 ± 11.4^a^	102 ± 10.6^b^
(*Z*)-3-Hexen-1-ol	1389	858	312 ± 17.2^a^	23 ± 2.0^b^
Hexan-1-ol	1079	872	60 ± 3.5^a^	33 ± 1.5^b^
2,6-Dimethylcyclohexanol	1112	979	tr	tr
1-Octen-3-ol	1451	979	tr	tr
2-Phenylethanol	1911	1117	2457 ± 151.0^a^	97 ± 5.9^b^
Aldehydes				
2-Phenylethanal	1037	–	tr	21 ± 2.1^a^
Benzaldehyde	1524	1517	tr	35 ± 3.2^a^
Acids				
2-Ethyl hexanoic acid	1129	–	tr	Nd
Acetic acid	1428	600	18 ± 2.7^a^	19 ± 0.8^a^
Esters				
Ethyl-2-methylpropionate	961	758	315 ± 26.0	Nd
Methylbutanoate	981	723	205 ± 16.0^a^	tr
Ethylbutanoate	1028	803	604 ± 112.0	Nd
1-Pentyl acetate	1170	919	37 ± 4.3	Nd
Methyl hexanoate	–	1000	433 ± 45.1	Nd
Butyl butanoate	1218	995	65 ± 5.6	Nd
2-Heptyl acetate	1259	1040	tr	tr
Hexyl acetate	1270	1014	522 ± 101.6	Nd
(*Z)*-3-Hexenyl acetate	1325	1007	125 ± 2.5^a^	tr
Methyl octanoate	–	1137	475 ± 96.0^a^	35 ± 1.5^b^
Ethyl cinnamate	2167	1469	715 ± 117.0	Nd
Terpenes				
Limonene	1185	1030	127 ± 9.3	Nd
(E)-β-Ocimene	1250	1156	tr	Nd
Borneol	1253	885	tr	tr
(*Z)*-Rose oxide	1337	–	40 ± 5.0	Nd
(*E*)-α-Bergamotene	1415	–	tr	Nd
Linalool	1540	1103	5121 ± 107.0^a^	506 ± 19.4^b^
α-Terpineol	1582	1195	216 ± 5.0^a^	57 ± 6.7^b^
Geranial	1715	1277	114 ± 4.5	Nd
Geraniol	1840	–	341 ± 13.4^a^	79 ± 8.6^b^
Ketones				
Acetophenone	1067	–	42 ± 6.0^b^	437 ± 15.6^a^
4-Hydroxy-2,5-dimethyl-3(2H)-furanone	2038	1070	50 ± 2.6^b^	326 ± 15.0^a^
ϒ-Jasmolactone	2176	–	Nd	186 ± 11.7
Phenol				
Guaiacol	1842	1089	Nd	231 ± 14.3
Norisoprenoids				
Theaspirane isomer I	1280	–	Nd	tr
Theaspirane isomer II	1308	–	Nd	tr
β-Damascenone	1801	1389	tr	21 ± 1.7^a^
4-Hydroxy-β-ionol	1601	–	Nd	162 ± 10
β-Ionone	1933	1491	260 ± 12.0^a^	tr^b^
3-Oxo-α-ionol	1938	–	Nd	100 ± 12.5
4-Oxo-β-ionol	1943	–	Nd	141 ± 7.9
		Total	13,900 µg kg^−1^	3236 µg kg^−1^
		Alcohols	29.1%	26.1%
		Esters	25.2%	1.36%
		Terpenes	43%	20.1%
		Ketones	0.66%	29.3%
		Nop.	1.91%	13.3%

Mean ± SD (n = 3) with different superscript along the same row are significantly different (P < 0.05)

LR1, DB-FFAP; LR2, SE-54; *tr* trace amount (<10 µg kg^−1^), *Nd* not detected, *Nop* norisoprenoids

*LRI* linear retention index on column 1, *LR2* linear retention index on column 2

^1^Compounds were identified by comparing their retention indices on DB-FFAP and SE-54 columns, their mass spectra, and odour notes were compared with their respective reference odorants’ data

In the free fraction of the sweet black plum, the major aroma-active compounds (>300 µg kg^−1^) were linalool, 2-phenylethanol, 3-methyl-but-3-en-1-ol, ethyl cinnamate, ethylbutanoate, hexyl acetate, methyl octanoate, methyl hexanoate, ethyl-2-methylpropionate, geraniol, and (*Z*)-3-hexen-1-ol. These compounds accounted for 88.8% of the aroma in the free fraction. In addition, most of these compounds were previously reported in several fruits such as lychee, strawberry, cherry and oranges [8, 12–14] either in the free or bound form. The identification of significant numbers of fatty acid esters such as methylbutanoate, ethylbutanoate and methyl hexanoate is an indication of the possible contribution of lipid metabolism in the biogenesis of Vds aroma. Volatile esters are produced by virtually all fruit species during ripening. Most volatile esters have flavour characteristics described as fruity [15]. Worthy of note was the high concentration of linalool (5121 µg kg^−1^) in the Vds. This floral-like terpene alcohol which is produced from isopentenyl pyrophosphate via the universal isoprenoid intermediate geranyl pyrophosphate, and membrane-bound enzymes such as linalool synthase [16] has been reported in lychee [17], Coastal Rican guava [18], mangaba fruit [19] and black velvet tamarind [20]. Another compound of interest is the honey-like 2-phenyl ethanol which produced a significant concentration in the free fraction. The odorant is an important flavour compound in the food and cosmetic industries.

The major volatile compounds in the bound fraction of the Vds were; 4-hydroxy-β-ionol, guaiacol, *y*-jasmolactone, 4-hydroxy-2,5-dimethyl-3(2H)-furanone, acetophenone, linalool and 3-methyl-but-3-en-1-ol (Table 1). In comparison to the free volatile compounds, which were mainly alcohols, esters and terpenes, the bound volatiles profiles included alcohols, ketones, and norisoprenoids. While most of the alcohols detected in the free fraction, were found in the bound form, there were fewer esters identified in the bound form. Only methyl octanoate was detected in both fractions. The reason for this observation is not farfetched because glycosidically bound volatiles are organic compounds in which the aglycone is volatile. This aglycone must be bounded to the sugar via ‘glycosidic bond’, for which these compounds have to have an –OH–, –SH, or –NH. Thus aldehydes, esters and terpenes are not able to form glycosidical bonds. Although, similar alcohol profiles were obtained from both free and bound fractions, the concentrations of the alcohols in the bound fraction were significantly (P < 0.05) lower to that of the free fraction. Of interest is the high abundance of 3-methyl-but-3-en-1-ol in both fractions. The presence of this compound in the bound form attested to the fact that it is an important intermediate in various biosynthetic pathways. In addition, significant numbers of odorous norisoprenoids were detected in the bound fraction. Among them were the floral 4-hydroxy-β-ionol, the spicy 3-oxo-α-ionol, 4-oxo-β-ionol and the flowery β-damascenone. Most of these compounds have been detected in several fruits such as grape [21], apple [22], raspberry [11] and passion fruit [23]. Also, identified in trace amounts (<10 µg kg^−1^) in the bound fraction were the two isomers (I & II) of theaspirane.

However, to gain an insight into the contribution of the aroma compounds to the aroma notes of the free and bound fractions, the 36 odorants detected through aroma extract dilution analysis (AEDA) as the key odorants were quantified. The flavour dilution (FD) factors obtained for the key odorants ranged from 2 to 512 (Table 2). Results revealed an array of aroma notes as shown in Table 2. The seventeen odorants with FD factors ≥16 were further investigated. The results of the quantitation showed that linalool was the predominant compound in both the free (5121 µg kg^−1^) and the bound (506 µg kg^−1^) fractions respectively (Table 3). This was followed by 2-phenyl ethanol (2457 µg kg^−1^) in the free fraction and acetophenone in the bound fraction. However, a comparative analysis of the aroma potencies revealed that the free volatile fraction of the Vds exhibited more potency for the ethyl-2-methylpropionate, β-damascenone and ethylbutanoate as exemplified by their high odour activity values (OAVs) (Table 3). On the other hand, the bound fraction recorded higher OAVs for β-damascenone and linalool respectively. Also, the OAVs indicated that hexyl acetate, ethyl-2-methylpropionate, ethylbutanoate, linalool, β-damacenone and (*Z*)-rose oxide contributed to the sweet prune-like aroma of the Vds. Interestingly, compounds with high concentration such as 2-phenyl ethanol (2457 µg kg^−1^), geraniol and methyl butanoate gave low OAVs. Therefore, their contribution to the aroma note of the Vds can be assumed to be low.

**Table 2:** Key odorants (free and bound) detected in *Vitex doniana* sweet

No	Compound	Odour impression	DB-FFAP	FD
1	Ethyl-2-methylpropionate^a^	Fruity	961	32
2	Methylbutanoate^a^	Fruity	981	128
3	Ethylbutanoate^a^	Banana-like	1028	16
4	2-Phenylethanal^b^	Honey-like	1037	4
5	Acetophenone^a^	Cherry-like	1067	512
6	Hexan-1-ol^a^	Green, blooming	1079	2
7	2,6-Dimethylcyclohexanol^c^	–	112	Nd
8	2-Ethyl hexanoic acid^a^	–	1129	Nd
9	1-Pentyl acetate^a^	Herbal-like	1170	2
10	Limonene^a^	Orange-like	1185	16
11	3-Methylbut-3-en-1-ol^a^	Slightly apple-like	1209	8
12	2/3-Methylbutanol^a^	Solvent	1213	4
13	Butyl butanoate^a^	Fruity, pineapple	1218	32
14	(*E*)-β-Ocimene^b^	Flowery, blooming	1250	64
15	Borneol^b^	Camphor-like	1253	2
16	2-Heptyl acetate^a^	Woody, rum-like	1259	2
17	Hexyl acetate^a^	Fruity	1270	16
18	(Z)-3-Hexenyl acetate^a^	Fresh, pear-like	1337	8
19	(Z)-Rose oxide^a^	Rose-like	1337	16
20	(Z)-3-Hexen-1-ol^a^	Green	1389	8
21	(E)-α-Bergamotene^b^	floral	1415	8
22	Acetic acid^a^	Sweaty	1428	4
23	1-Octen-3-ol^a^	Mushroom-like	1451	2
24	Benzaldehyde^a^	Almond-like	1521	16
25	Linalool^a^	Flowery	1540	16
26	α-Terpineol^a^	Floral	1582	8
27	4-Hydroxy-β-ionol^a^	Floral	1601	16
28	Geranial^a^	Rose-like	1715	8
29	β-Damascenone^a^	Flowery	1801	16
30	Geraniol^a^	Rose-like	1840	16
31	Guaiacol^a^	Smoky	1842	4
32	2-Phenylethanol^a^	Honey-like	1911	16
33	β-Ionone^a^	Floral, violet-like	1933	4
34	3-Oxo-α-ionol^c^	Spicy	1938	2
35	4-Hydroxy-2,5-dimethyl-3(2H)-furanone^a^	Caramel-like	2038	16
36	Ethyl cinnamate^a^	Flowery, sweet	2167	32

*Nd* not determined, *FD* flavour dilution

^a^GC retention and MS data in agreement with that of the reference odorants

^b^GC retention and MS data in agreement with spectra found in the library

^c^Tentatively identified by MS matching with library spectra

**Table 3:** A comparative analysis of the aroma potency of compounds with flavour dilution (FD) values ≥16 in *Vitex doniana* sweet

No	Compounds	Conc.(µg kg^−1^fresh fruit) of fractions		Threshold (µg kg^−1^ of H_2_O) [ref.]	OAVs	
		Free	Bound		Free	Bound
1	Ethyl-2-methylpropionate	315	Nd	0.1 [4]	3150	Nd
2	Methylbutanoate	205	<10	28 [4]	7	<1
3	Ethylbutanoate	604	Nd	5 x 10^−2^ [4]	120,800	Nd
4	Acetophenone	42	437	65 [5]	<1	7
5	Limonene	127	Nd	210 [1]	<1	Nd
6	Butylbutanoate	65	Nd	100 [2]	<1	Nd
7	(E)-β-Ocimene	<10	Nd	–	Nd	Nd
8	Hexyl acetate	522	Nd	2 [4]	261	Nd
9	(Z)-Rose oxide	40	Nd	0.5 [1]	80	Nd
10	Benzaldehyde	<10	35	350 [5]	<1	<1
11	Linalool	5121	506	15 [3]	341	34
12	4-Hydroxy-β-ionol	Nd	162	–	Nd	Nd
13	Geraniol	79	341	40 [4]	2	9
14	β-Damascenone	<10	26	2 x 10^−3^ [4]	5000	10,500
15	2-Phenylethanol	2457	97	1000 [4]	3	<1
16	4-Hydroxy-2,5-dimethyl-3(2H)-furanone	50	326	40 [4]	1	8
17	Ethyl cinnamate	715	Nd	–	Nd	Nd

*Nd* not detected, *OAVs* odour activity values

[1] Maarse [29], [2] Takeoka et al. [30], [3] Lasekan & Ng [20], [4] Rychlik et al. [31], [5] Buttery et al. [32]

OAVs, calculated by dividing concentration with threshold value in water

**Fig. 2:**
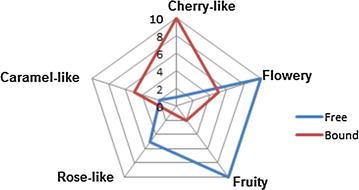
Comparative aroma profiles of bound and free compounds in *Vitex doniana* sweet

Sensory evaluation of both bound and free odorants of *V. doniana* sweet revealed distinct aroma characteristics. For instance, while the free fraction was characterised by the flowery and fruity notes, the bound fraction exhibited cherry-like, flowery, and caramel notes (Fig. 2). However to determine which compounds are responsible for the perceived aroma notes, a more detailed analysis on aroma models and omission test will be required.

## Conclusion

The study has revealed for the first time the aroma profiles of the free and glycosidically bound fractions of *V. doniana* sweet. In the free fraction, the predominant compounds were the terpenes, alcohols and esters. The glycosidically bound fraction was composed of ketones, alcohols, terpenes and norisoprenoids. Results of the OAVs revealed that while the free volatile fraction of the V*. doniana* sweet exhibited strong potency for the fruity and floral notes; the bound volatile fraction produced more of flowery, caramel and cherry-like notes. In addition, results have shown that ethylbutanoate, β-damascenone, ethyl-2-methyl propionate, linalool, hexyl acetate and (Z)-rose oxide contributed highly to the sweet prune-like aroma of *V. doniana* sweet.

## Materials and methods

### Fruit material

**Fig. 3:**
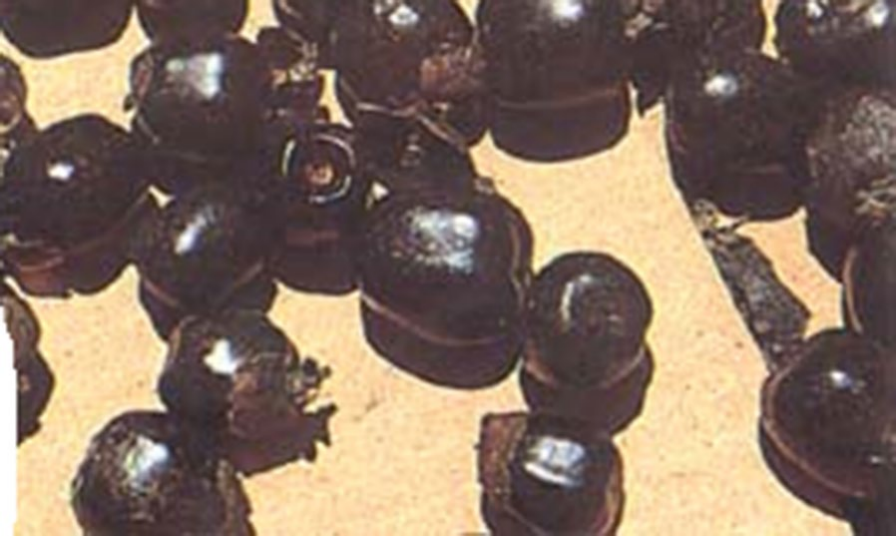
Ripened *Vitex doniana* sweet

Freshly harvested ripe *Vitex doniana* sweet (purple–black in colour) (Fig. 3) (300 fruits) grown in Owo, southwest Nigeria, were purchased from a local producer and stored (20 °C, 85% RH). The fruits were 2.8–3.2 cm in length, 1.2–1.4 cm in width and contained one hard conical seed each which is about 1.5–2.0 cm long and 1.0–1.2 cm wide. Quartering method [24] was used to select fruits for aroma analysis. At harvest, fruit had 10.5^o^ brix and a titratable acidity of 0.86% malic acid equivalent.

#### Reagents and standards

Ethanol, methanol and dichloromethane were purchased from Merck (Darmstadt, Germany), while sodium dihydrogen phosphate-1-hydrate,l- (+) -ascorbic acid, and citric acid were obtained from Panreac (Barcelona, Spain). Sodium fluoride and ethyl acetate were purchased from Fluka (Buchs, Switzerland). Almond β-glucosidase was obtained from Sigma Chemical (St. Louis, MO). Amberlite XAD-2 resins were purchased from Sigma-Aldrich (Poole, Dorset, UK) and pure water was from a Milli-Q purification system (Millipore, Bedford, MA, USA). An alkane solution (C_8_–C_24_; 20 mgL^−1^ dichloromethane) was used to calculate the linear retention index (LRI) for each analyte. Other reagents were of analytical grade.

The following reference chemicals: Acetic acid, methyl butanoate, ethyl-2-methyl propionate, ethyl butanoate, 2-ethylhexanoic acid, 3-methylbutanol, (*Z*)-3-hexen-1-ol, hexanol, octen-3-ol, benzaldehyde, 3-methyl-but-3-en-1-ol, 2-phenylethanol, 1-pentyl acetate, limonene, 3-methylbut-3-en-1ol, acetophenone, butylbutanoate, (*E*)-β-ocimene, 2-heptyl acetate, hexyl acetate, (*Z*)-3-hexenyl acetate, (*Z*)-rose oxide, (*Z*)-3-hexenol, (*E*)-α-bergamotene, 1-octen-3-ol, linalool, α-terpineol, 4-hydroxy-β-ionol, geranial, geraniol, guaiacol, β-damascenone, β-ionone, 4-hydroxy-2,5-dimethyl-3(2*H*)-furanone, ethylcinnamate were from Sigma-Aldrich (St. Louis, MO). Stock standard solutions of 10^3^ or 10^4^ µg mL^−1^ of each compound was prepared as described earlier [25].

### Fractionation of free aroma compounds of sweet black plum

Fruit pulp (500 g) was blended with 700 mL of distilled water. After 30 s, the mixture was centrifuged at 3000×*g* and 4 °C for 15 min. The supernatant was filtered through a bed of Celite. The clear Vds juice (300 mL) was applied onto an Amberlite XAD-2 adsorbent in a (30 × 2 cm) glass column. The column was washed with 250 mL of deionised water and 200 mL of n-pentane/diethyl ether mixture (1/1 v/v). The eluted extract was dried over anhydrous sodium sulphate and concentrated to 1 mL [26]. The concentrated extract (i.e. free fraction of the sweet black plum) was used for the GC–MS and GC–O analyses. The experiment was carried out in triplicate.

### Bound aroma compounds of the *V. doniana* sweet

After the free fraction was obtained from the Amberlite XAD-2 glass column, the glycosidic extract adsorbed on the column was collected by washing it with 250 mL of methanol. The obtained extract was dried over anhydrous sodium sulphate and similarly concentrated as the free fraction. The concentrated bound fraction was re-dissolved in 100 mL of phosphate-citrate buffer (0.2 M, pH 5.0) and washed (2×) with 45 mL of n-pentane/diethyl ether (1/1, v/v) to remove any free fraction. One mililiter of an almond β-glucosidase solution (5 unit mg^−1^ solid, concentration of 1 unit mL^−1^ buffer) was added to the glycosidic extract and incubated overnight at 37 °C [27]. The liberated aglycones were extracted with 30 mL of n-pentane/diethyl ether (1/1, v/v) (2×). The combined extracts were dried over anhydrous sodium sulphate, filtered and concentrated as described earlier [26]. The concentrated extract was used for the GC–MS analysis and the experiment was carried out in triplicate.

### GC–MS and GC–FID analyses

A Shimadzu (Kyoto, Japan) QP-5050A GC–MS equipped with a GC-17 A Ver.3, a flame ionization detector (FID) and fitted differently with columns DB-FFAP and SE-54 (each, 30 m × 0.32 mm i.d., film thickness 0.25 µm; Scientific Instrument Services, Inc., Ringoes, NJ) was employed. The gas chromatographic and mass spectrometric conditions were the same as described previously by Lasekan & Ng, [20]. The HP Chemstation Software was employed for the data acquisition and mass spectra were identified using the NIST/NB575K database.

### Gas chromatography–olfactometry

A Trace Ultra 1300 gas chromatograph (Thermo Scientific, Waltham, MA, USA) fitted with a DB-FFAP column (30 m × 0.32 mm i.d., film thickness, 0.25 µm, Scientific Instrument Services, Inc., Ringoes, NJ) and an ODP 3 olfactory Detector Port (Gerstel, Mulheim, Germany), with additional supply of humidified purge air, was operated as earlier reported by Lasekan et al. [25]. The split ratio between the sniffing port and the FID detector was 1:1. Two replicate samples were sniffed by three trained panellists who presented normalised responses, reproducibility and agreement with one another. The GC–O analysis was divided into three parts of 20 min and each panellist participated in the sniffing. An aroma note is valid only when the three panellists were able to detect the odour note.

### Identification and quantification

The linear retention indices were calculated according to Kovats method using a mixture of normal paraffin C_6_–C_28_ as external references. The identification of volatiles was carried out by comparing their retention indices, mass spectra data and odour notes with those of the reference odorants, literature data or with the data bank (NIST/NB575K). Quantitative data were obtained by relating the peak area of each odorant to that of the corresponding external standard and were expressed as µg kg^−1^.

### Aroma extracts dilution analysis (AEDA)

The extracts of the free and bound fractions were diluted step wise twofold with dichloromethane by volume to obtain dilutions of 1:2, 1:4, 1:8, and 1:16 and so on. Each obtained dilution was injected into the GC–O. The highest dilution in which an aroma compound was observed is referred to as the FD factor of that compound [28].

### Aroma profile determination

Fresh Vds (40 g) were placed inside glass containers (7 cm × 3.5 cm) and were orthonasally analysed as described earlier [20]. Reference odorants used were: Acetophenone (cherry-like), linalool (Flowery), (*Z)*-rose oxide (rose-like), 4-hydroxy-2,5-dimethyl-3(2H)-furanone (caramel-like) and hexyl acetate (fruity). Panellists rated the intensities of each descriptor on an unstructured scale from 0 to 10, where 0 = not detectable, 5 = weak, and 10 = strong. Final results were presented in a web plot.

### Statistical analysis

Statistical analyses were carried out with SPSS version 16.0 Windows (SPSS Inc., Chicago, IL). Significance of differences between means was tested by one-way analysis of variance (ANOVA). Results were expressed as mean ± SD (standard deviation) of triplicate analyses.
